# Autophagy of glycogen is non-selective in *Komagataella phaffii*

**DOI:** 10.1080/27694127.2024.2382659

**Published:** 2024-07-27

**Authors:** Taras Y. Nazarko

**Affiliations:** Department of Biology, Georgia State University, Atlanta, USA

**Keywords:** Autophagy, Glg1, glycogen, glycogenin, glycophagy, *Komagataella phaffii*, yeast

## Abstract

Glycogen is an important reserve polysaccharide from bacteria to human. It is organized in glycogen granules that also contain several proteins involved in their metabolism. Glycogen granules can be mobilized in mammalian lysosomes and yeast vacuoles. They are delivered to these organelles by macroautophagy (hereafter autophagy). However, whether this is a selective or a non-selective process remains a matter of debate. It was proposed to be selective and called “glycophagy” (for selective autophagy of glycogen) in mouse liver. However, the evidence of this selectivity is lacking in other glycogen-rich organs, such as the heart and skeletal muscle, which both are heavily impacted by the aberrant lysosomal accumulation of glycogen in Pompe disease. We recently developed the *Komagataella phaffii* yeast as a simple model to study the relationship of glycogen and autophagy. Using this model, we showed that cytosolic glycogen granules are delivered to the vacuole by non-selective autophagy, at least during nitrogen starvation. We speculate that this type of autophagy might be responsible for the lysosomal glycogen turnover in non-hepatic mammalian tissues.

**Abbreviations:**

GFP, green fluorescent protein.

Glycogen is a ubiquitous polysaccharide and the main storage form of glucose in many cell types. Glycogen is synthesized by several proteins that create α-1,4- and α-1,6-glycosidic bonds between glucose residues to make this branched polymer. The first protein in the pathway of glycogen biosynthesis is glycogenin (Glg1 in *K. phaffii*). This enzyme first glucosylates its own tyrosine residue via the glucose-1-*O*-tyrosyl linkage and then, adds several more glucose residues to the initiated chain via α-1,4-glycosidic bonds. As such, the growing molecule of glycogen is from the very beginning covalently bound to the molecule of glycogenin that has nucleated it. Subsequently, the glycogen synthase takes over the elongation process and adds many more residues of glucose through the same α-1,4-glycosidic linkage before the branching enzyme moves terminal stretches of glycogen to its side and attaches them through α-1,6-glycosidic bonds. As glycogen grows and branches, other enzymes and regulatory proteins become associated with it creating what is called a glycogen granule.

Glycogen granules are small and numerous. They are the most abundant in cells of the liver, the heart and skeletal muscles. During starvation, cells quickly catabolize glycogen to glucose-1-phosphate by the cytosolic glycogen phosphorylase. In addition, they also deliver entire glycogen granules to lysosomes/vacuoles for hydrolysis by the lysosomal acid α-glucosidase. The lack of the latter enzyme causes the lysosomal storage disorder called Pompe disease, which is characterized by the accumulation of glycogen granules in lysosomes and affects the functionality of the heart and skeletal muscles. Therefore, it is important to understand the mechanism of glycogen delivery to lysosomes. While several studies established macroautophagy (hereafter autophagy) and autophagosomes as being responsible for this transport, the type of autophagy remained obscure. This question is particularly important in the heart and skeletal muscles where the autophagy of glycogen granules proceeds independently of Stbd1 (starch binding domain 1) protein, which was proposed to be the selective autophagy receptor of glycogen in mouse liver.

In our recent study [1], we clarified what type of autophagy is responsible for the degradation of glycogen in nitrogen-starved *K. phaffii*. The *K. phaffii* yeast is a well-established autophagic model. In our work [1], we developed *K. phaffii* as a new model to study the glycogen granule homeostasis. For this, we first confirmed that *K. phaffii* Glg1 is indeed essential for glycogen biosynthesis. Then, we showed that the Glg1-GFP fusion is fully functional. As such and because of its covalent binding to glycogen, Glg1-GFP could be used as a marker protein for glycogen granules and a reporter protein for their cytoplasm-to-vacuole trafficking. Further fluorescence microscopy and western blotting experiments indicated that similar to more complex eukaryotes, glycogen granules in *K. phaffii* are delivered to the vacuole by autophagy highlighting the suitability of *K. phaffii* model to study glycogen autophagy type and mechanism.

There are two major types of autophagy, selective and non-selective. Most yeast selective autophagy pathways utilize the adaptor protein Atg11. Therefore, an autophagic pathway, which is Atg11-dependent, is very likely to be selective. We found that the autophagy of glycogen granules is independent of Atg11 in *K. phaffii* [1]. However, this did not exclude the possibility that glycogen autophagy could still be selective, like e.g. the selective autophagy of protein aggregates that requires the selective autophagy receptor Cue5 but not the adaptor Atg11. Therefore, we conducted additional experiments to address this issue. We compared the vacuolar delivery and turnover kinetics of the glycogen-bound Glg1-GFP, the cytosolic Glg1^Y212F^-GFP, a mutant variant that lacks a conserved tyrosine residue in the position 212 essential for Glg1 self-glucosylation and glycogen formation, and the cytosolic Pgk1-GFP, which is an established reporter for a non-selective autophagy. Importantly, both the delivery to the vacuole and vacuolar degradation of Glg1-GFP were not more efficient than those of two cytosolic reporters, indicating that the autophagy of glycogen granules is a non-selective process in *K. phaffii*.

All the autophagy experiments in our study were performed under nitrogen starvation conditions [1]. Therefore, we cannot exclude the possibility that other conditions, such as carbon starvation, might involve a more efficient, selective type of autophagy for glycogen (i.e. glycophagy) in *K. phaffii*. Moreover, similar studies in other yeasts are necessary to determine how widespread such a response to nitrogen starvation is across yeast species. Of particular interest are also the future studies in mice where glycogen was shown to be delivered to lysosomes by the Stbd1-mediated glycophagy in the liver, but via some Stbd1-independent autophagic pathway in the heart and skeletal muscles. Because our work in *K. phaffii* revealed that the autophagy of glycogen can be non-selective, further inquiry into the type of glycogen autophagy in non-hepatic mammalian tissues is now warranted.
